# Technology-enhanced multi-domain at home continuum of care program with respect to usual care for people with cognitive impairment: the Ability-TelerehABILITation study protocol for a randomized controlled trial

**DOI:** 10.1186/s12888-016-1132-y

**Published:** 2016-11-25

**Authors:** O. Realdon, F. Rossetto, M. Nalin, I. Baroni, M. Cabinio, R. Fioravanti, F. L. Saibene, M. Alberoni, F. Mantovani, M. Romano, R. Nemni, F. Baglio

**Affiliations:** 1 Department of Human Sciences for Education, Università degli Studi di Milano-Bicocca, Milan, Italy; 2 IRCCS, Fondazione don Carlo Gnocchi ONLUS, Via Capecelatro 66, 20148 Milan, Italy; 3 Department of Psychology, Università Cattolica del Sacro Cuore, Milan, Italy; 4 R&D, Telbios srl, Milan, Italy; 5 Department of Physiopathology and Transplants, Università degli Studi di Milano, Milan, Italy

**Keywords:** Telerehabilitation, Dementia, Mild cognitive impairment, Alzheimer’s disease, Continuum of care

## Abstract

**Background:**

According to the World Alzheimer Report (Prince, The Global Impact of Dementia: an Analysis of Prevalence, Incidence, Cost and Trends, 2015), 46.8 million people worldwide are nowadays living with dementia. And this number is estimated to approximate 131.5 million by 2050, with an increasing burden on society and families. The lack of medical treatments able to stop or slow down the course of the disease has moved the focus of interest toward the *nonpharmacological approach* and *psychosocial therapies* for people with/at risk of dementia, as in the Mild Cognitive Impairment (MCI) condition. The purpose of the present study is to test an individualized home-based multidimensional program aimed at enhancing the continuum of care for MCI and outpatients with dementia in early stage using technology.

**Methods:**

The proposed study is a single blind randomized controlled trial (RCT) involving 30 subjects with MCI and Alzheimer’s disease (AD) randomly assigned to the intervention group (Ability group), who will receive the “Ability Program”, or to the active control group (ACG), who will receive “Treatment As Usual” (TAU). The protocol provides for three steps of assessment: at the baseline (T_0), after treatment, (T_1) and at follow-up (T_2) with a multidimensional evaluation battery including cognitive functioning, behavioral, functional, and quality of life measures. The Ability Program lasts 6 weeks, comprises tablet-delivered cognitive (5 days/week) and physical activities (7 days/week) combined with a set of devices for the measurement and monitoring from remote of vital and physical health parameters. The TAU equally lasts 6 weeks and includes paper and pencil cognitive activities (5 days/week), with clinician’s prescription to perform physical exercise every day and to monitor selected vital parameters.

**Discussion:**

Results of this study will inform on the efficacy of a technology-enhanced home care service to preserve cognitive and motor levels of functioning in MCI and AD, in order to slow down their loss of autonomy in daily life. The expected outcome is to ensure the continuity of care from clinical practice to the patient’s home, enabling also cost effectiveness and the empowerment of patient and caregiver in the care process, positively impacting on their quality of life.

**Trial registration:**

ClinicalTrials.gov ID: NCT02746484 (registration date: 12/apr/2016 – retrospectively registered).

## Background

Dementia is now considered as a social emergency since it became one of the 25 main causes of disability in people worldwide over the last few decades [25]. Dementia affects multiple domains, such as cognitive functioning, behavioral aspects and functional skills. It has also a significant impact on personal autonomy in daily life and social relationships, associated to a high level of caregiver burden as indicated by data from 2015 World Alzheimer Report [28]. The most common form of dementia is Alzheimer’s disease (AD), a progressive neurodegenerative condition accounting for up to 60% of all dementia cases [38]. From the clinical and health management point of view, Mild Cognitive Impairment (MCI) and early stages of AD represent the most interesting clinical conditions. MCI is considered a condition at risk for the development of dementia, therefore early interventions can be essential to prevent or delay further decline. Currently, no pharmacological therapies are available to prevent a potential conversion from MCI to AD condition or to change the AD course. However, different types of non pharmacological interventions have been developed and an increasing body of evidence shows that cognitive stimulation programs bring benefits to cognitive function of people with mild to moderate dementia over and above any medication effects [6, 39]. Within the framework of non pharmacological interventions, the use of technology to assist the person at risk and/or with mild dementia at home and to extend rehabilitation services in the treatment of dementia has gradually gained importance. It provides a range of home services based on innovative technologies which include therapeutic interventions, remote monitoring of progress and training for caregivers of people with disability [18]. These long-distance rehabilitation services were primarily developed in order to provide equitable access to local sanitary systems for individuals who are geographically remote or with physical disabilities for whom it is difficult to reach the hospital [35]. Nowadays, as long as the potential benefits of telerehabilitation have been highlighted in the literature [2], these technologies are also being applied to cognitive rehabilitation. In fact, unlike usual face-to-face care rehabilitation protocols, these technological approaches appear to be more advantageous in terms of intensity and duration of treatment, lowest costs and support of dyad (patient and caregiver) at home [11, 20].

Nonetheless, the efficiency and efficacy of home-based technology-enhanced continuum of care programs are poorly examined and evidences are controversial. Moreover, functional skills, level of patient engagement at home and psychological well-being are under-investigated despite their being key outcomes of interest [21]. Randomized controlled trials (RCT) are strongly needed to determine the optimal design of home-based treatment protocols and identify their long-term benefits.

The main aim of the proposed trial is to test the performance of a technology-enhanced rehabilitation and telemonitoring program (The Ability Program) to ensure the continuity of care from clinical setting to patient’s home. We will investigate whether MCI and people with early AD participating in the Ability program will show improvements in cognition and quality of life (outcome evaluation; efficacy) and better adherence and engagement in the program (output evaluation; efficiency) with respect to MCI and AD subjects taking part in the TAU.

## Methods

### Study design

**Fig. 1:**
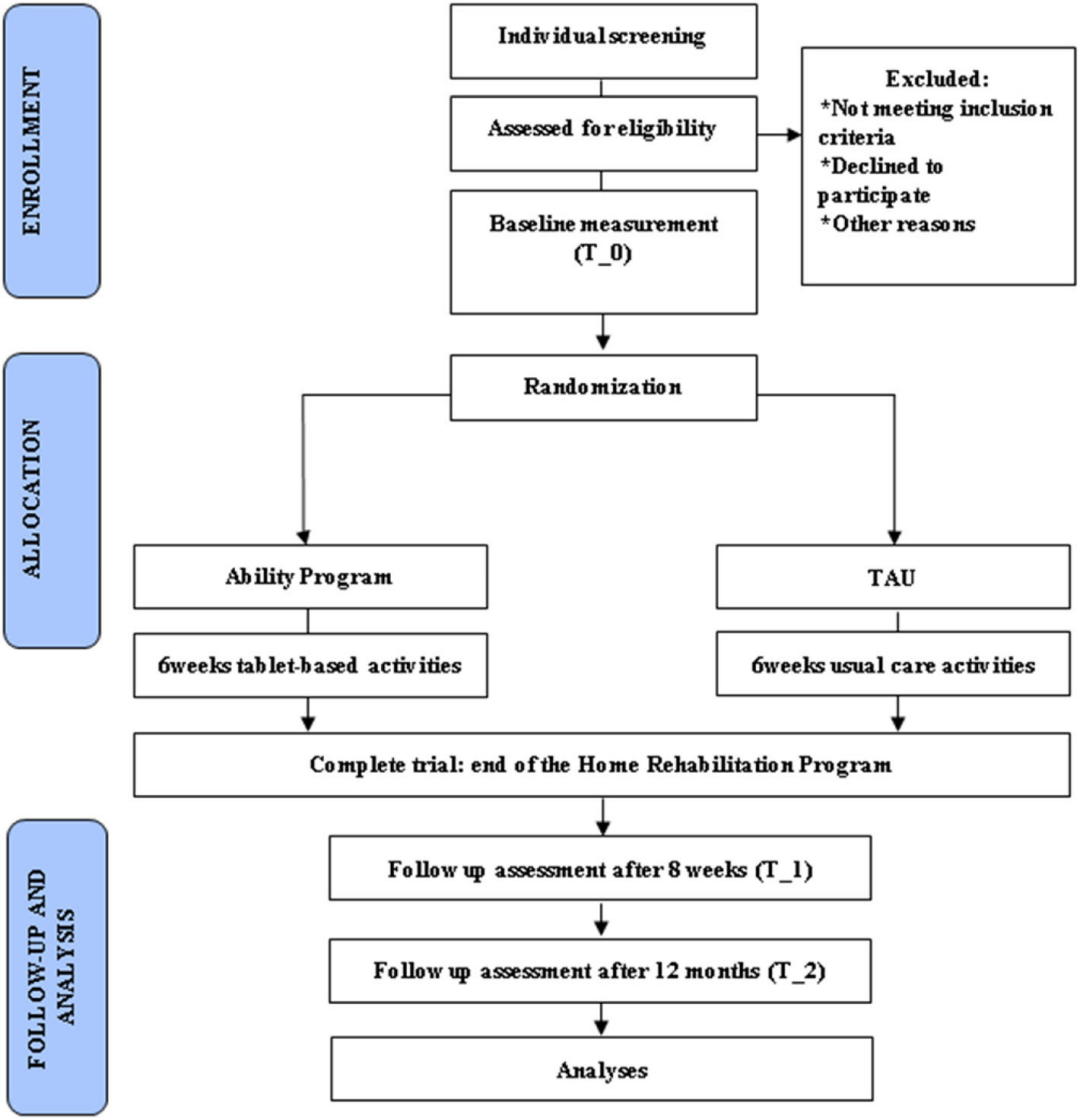
CONSORT flow diagram. Consolidated Standards Of Reporting Trials (CONSORT) flow diagram for enrollment and randomization in the TelerehABILITation study

The design is a single-blind, randomized, two-treatment arms (Ability Program, over 6 weeks vs. TAU, over 6 weeks) controlled clinical trial. After enrollment and baseline assessment, MCI and AD subjects will be randomized (with an allocation ratio of 1:1) into either the Ability group (30 motor-cognitive rehabilitation sessions combined with the measurement and monitoring from remote of vital parameters delivered at home through the Ability platform) or the active control group (ACG) (30 motor-cognitive rehabilitation sessions combined with the measurement of vital parameters to be performed at home through the usual care treatment procedure). Primary and secondary outcome measures will be obtained at baseline (T_0) before starting either program, post-intervention at 8 weeks after baseline (T_1) and at follow up 12 months after baseline (T_2). Consolidated Standards Of Reporting Trials (CONSORT) flow diagram for enrollment and randomization in the TelerehABILITation study is showed in Fig. 1.

### Sample size

According to previous multicenter controlled studies [3, 14], sample size calculation was performed considering that under the assumption of normal distribution of the scores (primary outcomes differences between groups) and considering α level of .05, a sample size of 30 subjects resulted in a power greater than 70% and was therefore considered as adequate for this trial. To avoid loss to follow-up and maximize the follow-up sample, standard procedures (regular phone contacts with participants) will be applied.

### Participants

Thirty participants will be consecutively recruited from the Memory Clinic of IRCCS Don Carlo Gnocchi Foundation.

Outpatients inclusion criteria will be the following: a) diagnosis of mild AD or MCI due to AD, according to DSM- 5 diagnostic criteria and AD/MCI diagnostic criteria [23, 1]; b) MMSE score >18 (Mini Mental State Examination, [15, 22]); c) aged over 65 years and d) with school attendance ≥3 years.

Exclusion criteria will be a) severe auditory and/or visual loss; b) overt severe behavioral disturbances. and c) recent (1 month or less before starting the program) introduction or dose modification of the following pharmacological treatments: cholinesterase inhibitor, memantine, antidepressant, or antipsychotic drugs. Low-dose benzodiazepines for insomnia will be allowed during the study.

### Randomization and masking

Randomization will occur after screening and baseline assessments. Subjects will be randomly assigned to the Ability group or to the ACG with a 1:1 allocation ratio. Randomization will be conducted by an independent operator neither ot involved in the assessment and/or treatment using a computer algorithm (http://www.graphpad.com/quickcalcs/randMenu/). Since participants cannot be blinded to their treatment allocation, they will be instructed not to discuss the nature of their intervention with the researchers in charge of the assessments. Outcome measures will be collected by researchers blinded to group allocation.

### Intervention

The study protocol predicts the random assignment of all participants to the Ability Program or to the TAU according to a single-blind parallel-group study approach.

Both programs last six consecutive weeks and combine cognitive and motor activities with the measurement of vital parameters, all to be performed at home. According to the Individual Rehabilitation Plan (IRP), the rehabilitation protocol in both programs comprises the performing of cognitive exercises for 5 days a week and light motor activities for 7 days a week. The selection of vital parameters and frequency of their measurement is set by the clinician, according to individual diagnosis.

#### The Ability program

The Ability program is a technology-enhanced version of the home-based usual care program (TAU), with the same duration, type of rehabilitation domains (motor and cognitive), dose of respective rehabilitation sessions, prescription for the monitoring of vital parameters, with the addition of a tracking device for physical activity and sleep, and monitoring from remote of all the activities performed by the participant.

Subjects assigned to this group are provided with the Ability kit, a set of technological devices including: a tablet for the delivery of the IRP; a sphygmomanometer for the detection of blood pressure; a pulse oximeter for the measurement of oxygen blood level and heart rate; a scale for the detection of body weight, and a FitBit Charge to track physical and sleep activity.

For all the 6-week intervention, the IRP is delivered through the tablet that, every day, after opening its user-friendly dashboard, shows the activities planned by the clinician for that day. Cognitive exercises are delivered for 5 days a week, adapted motor activity is proposed for 3 days a week, while light aerobic motor activity is suggested for the remaining 4 days. At least once a week a message on the dashboard recalls subjects to measure the vital parameters critical for their clinical condition, according to medical prescription. As regards the FitBit tracker, no tablet-based recalls are predicted since participants are asked to wear it continuously (24 h) for all the 6 weeks, except for very short interruptions to recharge it (about 2 hours once a week).

#### Tablet-based cognitive and motor activities in the Ability program

Tablet-based cognitive activities aim to reinforce multiple cognitive domains: attention, reasoning, procedural, semantic and autobiographic memory, executive functions, and visual-spatial skills (see Table 1). Activities within each cognitive daily session are sequentially framed in a way that they typically begin with a learning phase in which several stimuli are proposed. Then, they typically continue with activities that stimulate attention and other cognitive functions. Finally, a recall task is proposed. Each activity is structured into five levels of difficulty that adaptively increase on the basis of subject’s performance. The total time predicted for each daily cognitive session is about 20 min.

**Table 1:** Tablet-based cognitive activities

Main domain	Activities: examples of contents
Language	Reading and comprehension of a piece of newspaper article
	Word production from semantic category in a specific time
	Word production based on a specific letter
	Word production based on a specific syllable
	Word production from a semantic category, based on a specific letter
Memory	Recall of popular songs, films or actor’s biographies
	Memorization of daily life notes or brief messages and recall at the end of the cognitive session
	Memorization of a list of words and recall, searching for words which were not included in the list previously stored
	Recall of information in the newspaper article previously read and answering questions about it
	Carefully looking at a painting and memorizing
	Recall of information from a painting previously observed and answering some questions
	Inclusion of correct missing words in the text of some popular poems
Executive function	Putting things in the correct sequence based on a specific criterion
	Putting actions of daily life in the correct sequence
	Mathematical calculations
	Anagrams
	Rebuses (using pictures for words)
	Labyrinths
	Placing hands in empty clocks based on the time indicated in arabic figures
Attention	Finding specific words in a grid of mixed words
	Finding a specific letter in a grid of mixed letters

**Fig. 2:**
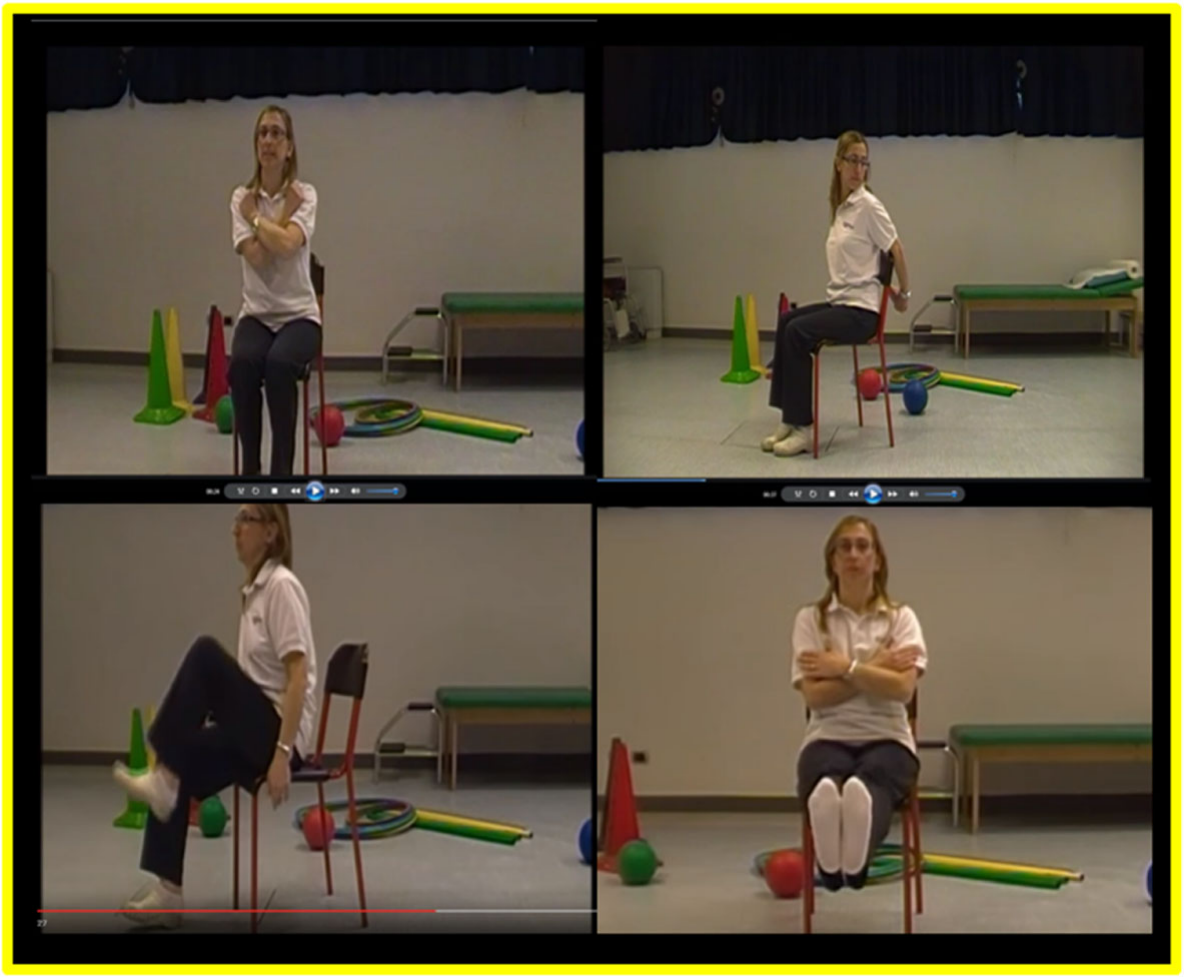
Motor activities in the Ability Program. Tablet-delivered motor activities in the Ability Program for the upper and lower arm, the trunk and the cervical spine, conducted by a physiotherapist and reported via video clips

Aerobic physical exercises are adapted motor activities [13] that involve the performing of simple movements, e.g., shoulder rotations, arm rotations, lower limb flexion, and neck, shoulder, or arm stretching. Exercises are reported via video clips (see Fig. 2). Each activity starts with a brief explanatory training conducted by the physiotherapist that also shows the correct execution of the exercises. Then the subject starts the movements mirroring the therapist while watching the video. Soft background music in time with the activity is played to improve the synchronized movement pattern, to capture attention and to foster engagement with the task [34]. Subjects use the support of a chair in all activities, both for home safety and since they often suffer from (sub) clinical balance disturbances. The total time envisioned for each adapted motor activity is about 15 min.

Before starting the program, participant and caregiver dyads are invited at the Memory Clinic to collect the Ability kit. On that occasion, they go through a training session lasting approximately two hours. The training that researchers will provide to participants and their carers will focus both on familiarizing with the technological devices of the kit and on how to use them. After a brief explanation of the functions of the several devices of the kit, researchers will conduct the training with a learning-by-doing experiential approach, scaffolding dyads in the management, from beginning to end, of a sample daily Ability session. The aim is to let patients and their carers get a working knowledge of the technological devices of the kit, meant for them as interfaces enabling their care experience, rather than focusing on abstract explanations on the technologies at hand. The participatory appropriation training starts with the opening of the tablet, the familiarization phase with the dashboard, and proceeds with a sample daily session of cognitive activities. The sample session, specifically devised for training purposes, includes at least one cognitive activity within each type of the different implementation designs developed. By implementation design we mean the different actions (e.g., touching a blank space to let the keyboard appear) that a user needs to do to operationalize his intentions (e.g. write a word in a semantic fluency activity) in order to reach the desidered goal. Dyads are invited to operate by themselves on the tablet and, in case of mistakes, an errorless approach will be adopted, in order to encourage their sense of coherence and to empower self-efficacy beliefs in following the tasks proposed. Then, upon completion of the cognitive session, they will manage the transition back to the dashboard with the opportunity to either watch a video of an adapted motor activity or go to the vital parameters measurement recall session, in order to gain confidence in moving back and forth within the platform. Whatever the route selected on which to do first, they will then be invited to do the other, so as to have the opportunity to manage video watching through the tablet and to try the different vital parameters measurement devices. At the end of the tablet session, researchers will show the dyad the FitBit tracker that the participant will have to wear. They will instruct him to wear it day and night for all the 6 weeks of treatment, providing brief hints on its use. To wrap up all the information shared, researchers will provide dyads with a manual showing screenshots of all the basic tablet procedures within daily sessions and pictures describing in detail how to use, to charge, or to wear the several devices of the kit. Moreover, dyads will be informed that phone contacts are planned during the 6 weeks of intervention, with the opportunity of being called or to call themselves both a Support Service Centre for technical problems (e.g. connectivity) and a neuropsychologist as care manager.

#### Treatment As Usual

The ACG will receive TAU, which, according to IRP, comprises: a) paper and pencil cognitive activities to be performed at home 5 days a week, covering the same domains as the Ability Program (See Table 1), with five levels of difficulty progressively increasing week by week independently from participant’s performance the week before; b) prescription to perform light aerobic motor activity 7 days a week (e.g. walking for 30 min.); c) prescription to measure specific vital parameters according to individual clinical condition.

Before starting the program, the participant and carer dyad are invited at the Memory Clinic to collect the workbook containing paper and pencil cognitive activities and written clinicians’ prescriptions about motor activities and measurement of vital parameters. Researchers will provide the dyad with a brief training including instructions a) on the number of days per week in which to perform the exercises, at the same time encouraging the respect for pause periods; b) on the need to respect the dose of daily rehabilitation activities; c) on how to manage down-motivating drawbacks (especially in case of loss of perceived balance between skills and challenge in cognitive activities).

### Assessment design and outcomes measures

**Fig. 3:**
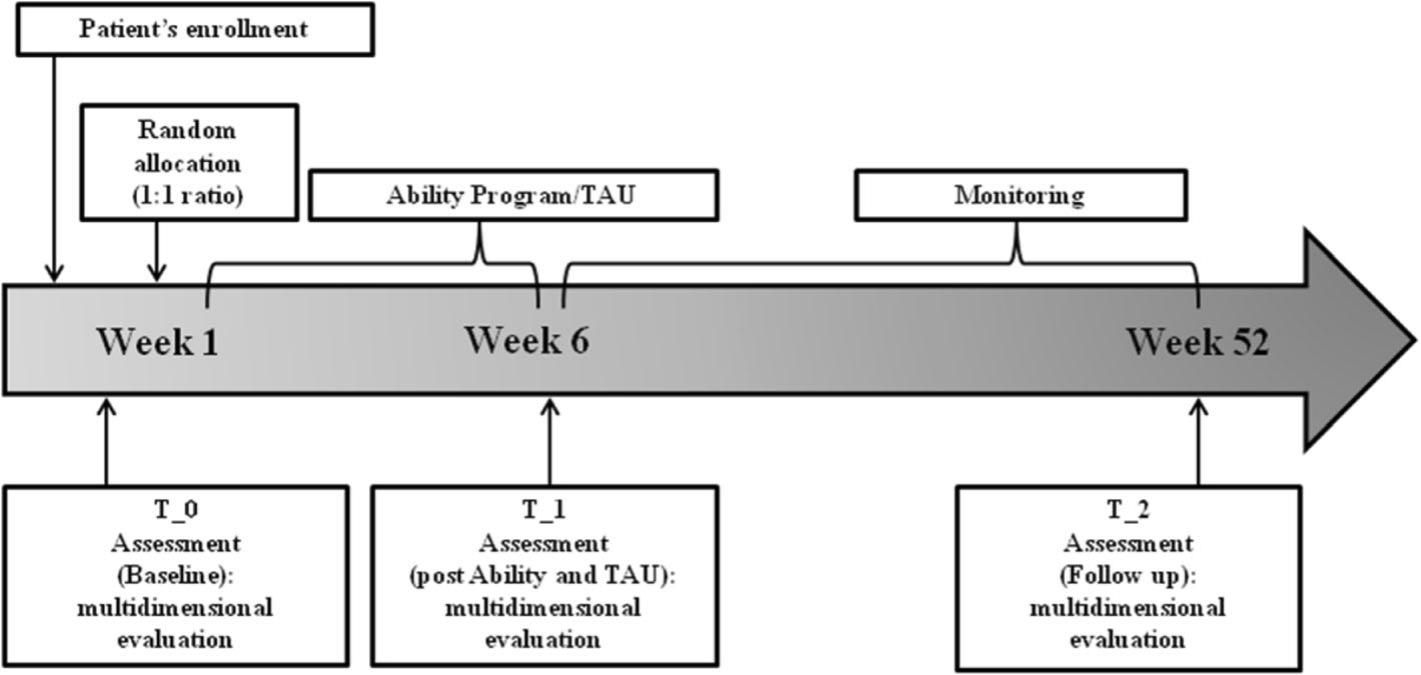
Timeline of the trial. Timing and duration of the various procedures in the trial design

The timeline of the study is illustrated in Fig. 3. Assessments will be done in both groups at baseline (T_0), at the end of intervention, 8 weeks after baseline (T_1), and at follow up, 12 months after baseline (T_2).

With respect to participants, we will adopt a multifaceted evaluation approach including cognitive functioning, behavioral, functional and perceived quality of life measures. Moreover we will also assess respective caregivers’ perceived burden and psychological well-being.

Participants’ cognitive functioning will be assessed through an extensive neuropsychological battery including:

- the Montreal Cognitive Assessment (MoCa; [9]), a sensitive tool for general cognitive level assessment;
- the Free and Cued Selective Reminding Test (FCSRT Delayed Free Recall (DFR) and Immediate Free Recall (IFR); [16]) for long-term memory evaluation;
- the Trail Making Test (TMT; [19]) for frontal-executive functions
- the Verbal Fluencies [27] for the assessment of language skills.

Participants’ functional and behavioral levels will be evaluated through the following proxy measures:

- the Activities of Daily Living Inventory (ADCS/ADL; [17])
- the Neuropsychiatric Inventory (NPI; [10]).

Both are proxy measures administered to caregivers.

Perceived participants’ quality of life (QoL) will be assessed through:

- the Dementia Quality of Life Instrument (D-QoL; [4]), a dementia-specific self-report measure recommended by the European consensus on outcome measures for non-pharmacological interventions on dementia [24],
- the Coping Orientation for Problems Experienced – Brief Version (Brief-COPE; [7]), since coping skills are considered as mediator variables for Quality of Life outcomes.

The evaluation of perceived burden and quality of life in caregivers will be done through:

- the Psychological Well-Being scale (PWB; [30, 31]);
- the Positive and Negative Affect Schedule (PANAS; [37, 33]);
- the Coping Orientation for Problems Experienced – Brief Version (Brief-COPE; [7, 32]);
- the Caregiver Burden Inventory (CBI; [26]).

Only participants in the Ability program group will be also administered, at the end of the intervention (T_1), the System Usability scale (SUS; [5]), in order to test how the Ability platform’s usability is perceived.

As primary outcome measure we consider the QoL of participants, being a measure of how a disease and its treatment affect a person’s capability to develop everyday activities and play valuable roles in their own life. Improvement in QoL plays a key role for individuals living with cognitive impairment, their family and carers. Secondary outcome measures include specific cognitive functioning such as memory, language and executive functions, being those abilities more compromised in MCI due to AD subjects [1], performance of complex instrumental activities of daily living, behavioral symptoms, and rates of conversion in dementia (see previous section).

### Statistical analyses

Statistical analyses on outcome measures will be conducted using SPSS. We will look at statistically significant changes in primary and secondary outcome measures from T_0 (start of treatment) to T_1 (end of treatment), i.e. a training effect. Moreover, we will look for a treatment (Ability vs. TAU) effect and at training x treatment interactions. This will be done through a 2-way ANOVA. Post hoc analysis will be applied to compare Ability with TAU. An alpha ≤ 0.05 will be considered significant. Moreover, score modification at T_2 (after 12 months) in both groups will be evaluated by means of regression analyses over time considering previous values at baseline and T_1. Contributing factors that may predict changing in outcomes will be also examined in a multivariate interval regression analysis. Moreover, each outcome will be categorized in three intervals according to the criterion of a clinical improvement/stability/impairment which was set by expert physicians. Given the exploratory nature of the study, a stepwise forward model will be set with an entrance alpha level of 0.10. Model assumptions and goodness of fit were evaluated by means of the proportionality of odds across response categories test and the association between predicted and observed values (Spearman rho). When the proportionality of odds assumption is violated, a generalized logistic regression model will be computed. For each significant model, several representative profiles and their associated probability of a successful treatment will be computed.

## Discussion and conclusions

In the framework of at home continuum of care programs, technologies have been increasingly conceived as a support for patients, their carers and healthcare professionals in the management of the disease. Increasing access to care, reducing health care costs and promoting the migration of care from medical institution to patient’s home are the principal aims of telerehabilitation for people with chronic conditions [12, 29].

In this context [36], the present innovative protocol is claiming to provide MCI subjects together with people with AD and their caregivers with an innovative technology-based program for the continuity of care in their favorite and most familiar environment, adopting a multidimensional rehabilitation approach including cognitive and motor activities. In this protocol, the Ability program is designed as an enhancement through technology of the usual care program, enabling additional relevant care options like scheduled delivery of the IRP, telemonitoring of vital parameters, and tracking of physical activity.

In order to minimize the lack of familiarity of elderly people with technologies, the challenge is to identify and to enhance individual factors that promote and facilitate human-technology interaction, increasing the acceptance of such an innovative and high-tech health care program [11]. To reach this goal, it is important to shape the intervention on the basis of user needs. As a novel telehealth platform, Ability is designed and implemented taking into account the patient’s age, education, interest, physical condition, access to technology, functional independence in daily life and the presence of supportive caregivers.

We expect that everyday rehabilitative sessions may have some positive effects on psychological and physical well-being of people with cognitive impairment, reducing cognitive and motor impairment and enhancing their quality of life. In addition, the Ability program may promote the continuity of care from clinical practice to patient’s home: it may be a more advantageous system for healthcare professionals, who have the opportunity to support the rehabilitation process in the distance, monitoring the progress and eventually changing the rehabilitation plan in a flexible and easy way according to a tailored intervention. The neurologist and the neuropsychologist are provided with the opportunity to check the rehabilitation plan execution at home from a dedicated platform contacting patients when appropriate (for example, in case of lack of compliance).

Caregivers can also have benefits from Ability program, which may result in the enhancement of their subjective perception of well-being and quality of life. We can assume that it may be useful to prevent or to mitigate caregiver burden by making the rehabilitation process easier to be carried out at home and by enhancing social interaction with the patient during the performance of the training program, promoting the quality of life of both patients and their caregivers through a joint engagement to reach the rehabilitation target [8]. Finally, we want to test the user experience of this program in elderly people, for which this practice is still often unfamiliar [40].

In sum, we expect that the Ability program can be a promising adaptive home-assistance service to contrast AD progression, testing its outcome in terms of rehabilitation, user experience and quality of life of people with MCI and early AD conditions and their carers.

### Trial status

The trial is ongoing.

## Abbreviations

ACG: Active control group
AD: Alzheimer’s Disease
ADCS/ADL: Activities of daily living
Brief-COPE: Coping orientation for problems experienced – brief version
CBI: Caregiver burden inventory
D-QoL: Dementia quality of life instrument
FCSRT: Free and cued selective reminding test
IRP: Individual rehabilitation plan
MCI: Mild cognitive impairment
MMSE: Mini mental state examination
MoCa: Montreal cognitive assessment
NPI: Neuropsychiatric inventory
PANAS: Positive and negative affect schedule
PWB: Psychological well-being scale
RCT: Randomized controlled trial
SUS: System usability scale
TAU: Treatment As Usual
TMT: Trail making test
